# An Internet-based Acceptance and Commitment Therapy intervention for older adults with anxiety complaints: study protocol for a cluster randomized controlled trial

**DOI:** 10.1186/s13063-018-2731-3

**Published:** 2018-09-17

**Authors:** Maartje Witlox, Vivivan Kraaij, Nadia Garnefski, Margot W. M. de Waal, Filip Smit, Erik Hoencamp, Jacobijn Gussekloo, Ernst T. Bohlmeijer, Philip Spinhoven

**Affiliations:** 1 Institute of Psychology, Leiden University, Leiden, The Netherlands; 2 Department of Public Health and Primary Care, Leiden University Medical Center, Leiden, The Netherlands; 3 Netherlands Institute of Mental Health and Addiction, Trimbos, Utrecht, The Netherlands; 4 Parnassia Psychiatric Institute, The Hague, The Netherlands; 5 Department of Public Health and Primary Care and Department of Gerontology and Geriatrics, Leiden University Medical Center, Leiden, The Netherlands; 6 Department of Psychology Health and Technology, University of Twente, Twente, The Netherlands

**Keywords:** Anxiety, Anxiety complaints, Subclinical anxiety, Older adults, Internet, Blended care, Acceptance and commitment therapy, Randomized controlled trial

## Abstract

**Background:**

Anxiety is among the most prevalent and disabling mental health problems in older adults. Few older adults with mild to moderately severe anxiety symptoms receive adequate interventions, putting them at risk for developing anxiety disorders, depression, and various somatic problems. Effective, low-threshold interventions should be developed. Blended care, in which a web-based intervention is combined with a limited amount of face-to-face contacts with a mental healthcare counselor at the general practice, is a promising option. The online self-help intervention “Living to the Full”—an Acceptance and Commitment Therapy (ACT) intervention—has been proven to reduce depression and anxiety in several patient groups, but has not yet been investigated in older adults. The aim of this study is to evaluate the (cost-)effectiveness of a blended form of “Living to the Full” in reducing anxiety symptoms in adults aged 55 to 75 years. Furthermore, moderators and mediators of the treatment effect are investigated.

**Methods/design:**

The (cost-)effectiveness of the ACT intervention will be investigated in a cluster single-blind randomized controlled trial (RCT). The blended intervention will be compared to treatment-as-usual. Thirty-six mental health counselors working at general practices in the Netherlands will be randomized to deliver blended care or treatment as usual. A total of 240 participants (aged 55–75 years) with mild to moderately severe anxiety complaints (defined as a total score of 5–15 on the GAD-7) will be recruited. There are four measurements consisting of online questionnaires (primary outcome: GAD-7) and a telephone interview: before the start of the intervention; directly following the intervention (14 weeks after baseline); and six and twelve months after baseline. Possible mediator variables will be assessed multiple times basis during the intervention.

**Discussion:**

This RCT will evaluate the effectiveness of a blended ACT intervention for older adults with anxiety symptoms. If the intervention is shown to be effective, it will be implemented, thereby improving the accessibility and quality of preventive interventions for older adults with anxiety problems.

**Trial registration:**

Netherlands Trial Register, NTR6270. Registered on 21 March 2017.

**Electronic supplementary material:**

The online version of this article (10.1186/s13063-018-2731-3) contains supplementary material, which is available to authorized users.

## Background

Anxiety problems form one of the most ubiquitous and disabling mental health conditions in older adults. Prevalence estimates of anxiety disorders in this age group are in the range of 1.2–14% [1, 2] . Furthermore, prevalence studies on anxiety in older adults consistently show that subclinical anxiety is even more widespread, with estimates in the range of 15–52.3% in community samples. These findings suggest that anxiety mostly presents as a sub-threshold disorder in elderly adults [1, 2]. Unfortunately, anxiety symptoms (and anxiety disorders as well) frequently go unrecognized and untreated in older adults [1–3]. This is due to several characteristics of this age group such as a stronger belief in self-reliance, higher perceived stigma of mental health problems, a limited willingness to accept treatment for such problems, a tendency to minimize symptoms, mobility problems, use of different terms to describe psychological problems, and problems with remembering and recognizing symptoms [4, 5]. Also, it seems challenging for both clinicians and patients to differentiate between functional and pathological anxiety in older adults; anxiety in elderly adults is regularly considered to be an epiphenomenon of a physical condition or part of the normal aging process [4, 6].

The fact that only a small proportion of anxious older adults receive adequate treatment is alarming, since even on a subclinical level, anxiety in older adults is associated with diminished quality of life and wellbeing [7, 8], depressive symptoms, hypertension, urinary incontinence [9], cognitive decline [10, 11], functional disability [12], and an increased risk for stroke [13] and coronary heart disease [14]. Furthermore, anxiety symptoms tend to run a chronic course [7] and put people at risk for developing anxiety disorders [15]. To diminish the personal and societal impact of anxiety complaints in older adults, low-threshold, evidence-based interventions are needed [15, 16]. However, considering the high prevalence of anxiety symptoms in older adults and the rise in life expectancy, even existing healthcare systems in affluent societies cannot adequately address this need. Internet-based therapy (possibly combined with a limited amount of face-to-face contacts with a therapist, counselor, or coach) is a promising option to reduce the treatment gap in a cost-effective way. For older adults with anxiety symptoms, Internet-based therapy could lower some of the barriers that prevent them from seeking help (e.g. perceived stigma on seeing a therapist, mobility problems, strong belief in self-reliance with regard to mental health).

In younger adult populations, Internet-based therapy—predominantly cognitive behavioral interventions—have repeatedly been shown to form a (cost-)effective treatment option for both anxiety disorders and subclinical anxiety [17–25]. Evidence so far suggests that a combination of an online self-help module and therapist support (either online, by telephone, or face-to-face) is the most effective form of Internet-based therapy [25].

To date, only one study has examined an online intervention for older adults with anxiety symptoms. This randomized controlled trial (RCT) found that Internet-delivered cognitive behavioral therapy (CBT) was an efficacious and cost-effective treatment for older adults with subclinical anxiety [26]. The vast majority of other trials in anxious older adults also focus on CBT but delivered as face-to-face therapy to patients with anxiety disorders. Several meta-analyses support the effectiveness of CBT for elderly adults with an anxiety disorder [27–31]. However, researchers suggest investigating other treatment approaches as well, since the effect sizes for CBT in anxious older adults are relatively small and several analyses suggest that older adults benefit less from CBT for anxiety than the younger adult population [2, 6, 28, 31, 32].

One treatment approach that might form a valuable alternative for CBT in older adults is Acceptance and Commitment Therapy (ACT), a so-called third-wave CBT [6, 33–35]. In contrast to CBT, ACT makes little to no use of content-oriented cognitive techniques. The goal of ACT is not to reduce the frequency or discomfort of negative internal experiences. Rather, ACT strives to reduce the struggle that arises when people try to suppress internal experiences and to stimulate people to engage in activities that are meaningful to them. Ultimately, ACT aims to promote psychological flexibility: the ability to fully and openly experience the present moment (including the negative or painful aspects); and to persist in or change behavior dependent on its accordance with personal goals and values [33, 35, 36]. Meta-analyses support the effectiveness of ACT in reducing various psychological problems, including anxiety disorders [36–39]. Internet interventions based on ACT have also been shown to be effective for adults with depressive and anxiety symptoms [40, 41]. No large-scale, controlled trials have yet been conducted to evaluate ACT interventions for older adults. However, it could be argued that the ACT approach might particularly resonate with older adults, as it concurs with the reorientation on important life values and associated value-directed behavior change in this life phase [34, 35]. Furthermore, an acceptance-based coping style could be especially valuable for older adults, because age-related adversities such as loss of dear ones and declining health might be most effectively coped with through an acceptance-oriented style. Some evidence suggests that in older adults, acceptance is associated with better emotional wellbeing and quality of life [35, 42–44]. Another possible benefit of ACT is its transdiagnostic approach, since anxiety and depression are highly co-morbid in older adults [1, 2, 45]. Some evidence suggests that co-morbid depressive symptoms have a negative impact on treatment outcome in anxious older adults (when treated with CBT) [46]. Therefore, a transdiagnostic treatment that focuses on psychological factors that might underlie both anxiety and depression is highly valuable.

Considering the need for evidence-based interventions for older adults with subclinical anxiety and the plausible suitability of ACT for this group, the proposed study aims to evaluate the (cost-)effectiveness of an Internet-based ACT intervention for older adults with anxiety complaints in general practice. To promote compliance and reduce dropout, this intervention is combined with a limited amount of face-to-face contacts with the mental health counselor at the general practice. This intervention will be compared to optimized treatment-as-usual. In addition to the analysis of the (cost-) effectiveness, moderators and mediators of treatment effects will also be investigated in the study. The current paper describes the research protocol for this study.

## Methods/design

### Design

The proposed study is a pragmatic cluster single-blind RCT. In a clustered trial, the unit of randomization is not the individual participant, but a group of participants (e.g. patients from one general practice, students from the same school, family members). In this study, randomization takes place on the level of the mental health counselors at general practices, which creates clusters of participants that receive treatment from the same counselor. Cluster randomization is used in this study to reduce experimental contamination [47] and to minimize administrative burden and time spent on training the participating mental health counselors in the used treatment methods. Participating mental health counselors will be randomly assigned to one of the following two conditions: they will either treat all included patients in their practice(s) with the Internet-based ACT intervention or provide all their patients with treatment-as-usual. In most cases, randomization of the mental health counselor will coincide with randomization of the practice(s) at which he/she is employed, but some practices employ multiple counselors. In these practices, a double randomization procedure will be used. First, the counselors are randomized. Second, when patients of this practice are included in the study, they are randomly allocated to one of the counselors in the practice.

The randomization table (containing 36 cells, block randomized into blocks of four cells) will be created by an independent researcher using *R* software. Participants, mental health counselors, and the main researcher (who serves as the contact person for the mental health counselors) cannot be blind for allocation to conditions. However, participants are not informed on whether the treatment they receive is the experimental or active control intervention. They are told that the study aims to compare two treatment options for people with mild to moderate anxiety complaints. This is considered an ethically sound approach, since treatment-as-usual is delivered as optimized care as usual.

Each participant will complete four main measurements during the study: before the start of the intervention (and before participants are informed about which intervention they will receive) (T0); directly following the intervention (three months after baseline; T1); and six (T2) and twelve months (T3) after baseline. All measurements consist mainly of online self-report questionnaires. T0, T1, and T3 also include a telephone interview conducted by a research assistant. The research assistant that conducts the telephone interviews will be unaware of treatment allocation of the participants. If for any reason this blinding is broken, another research assistant (that is blind to treatment condition of the participant) will take over the telephonic assessment.

**Fig. 1:**
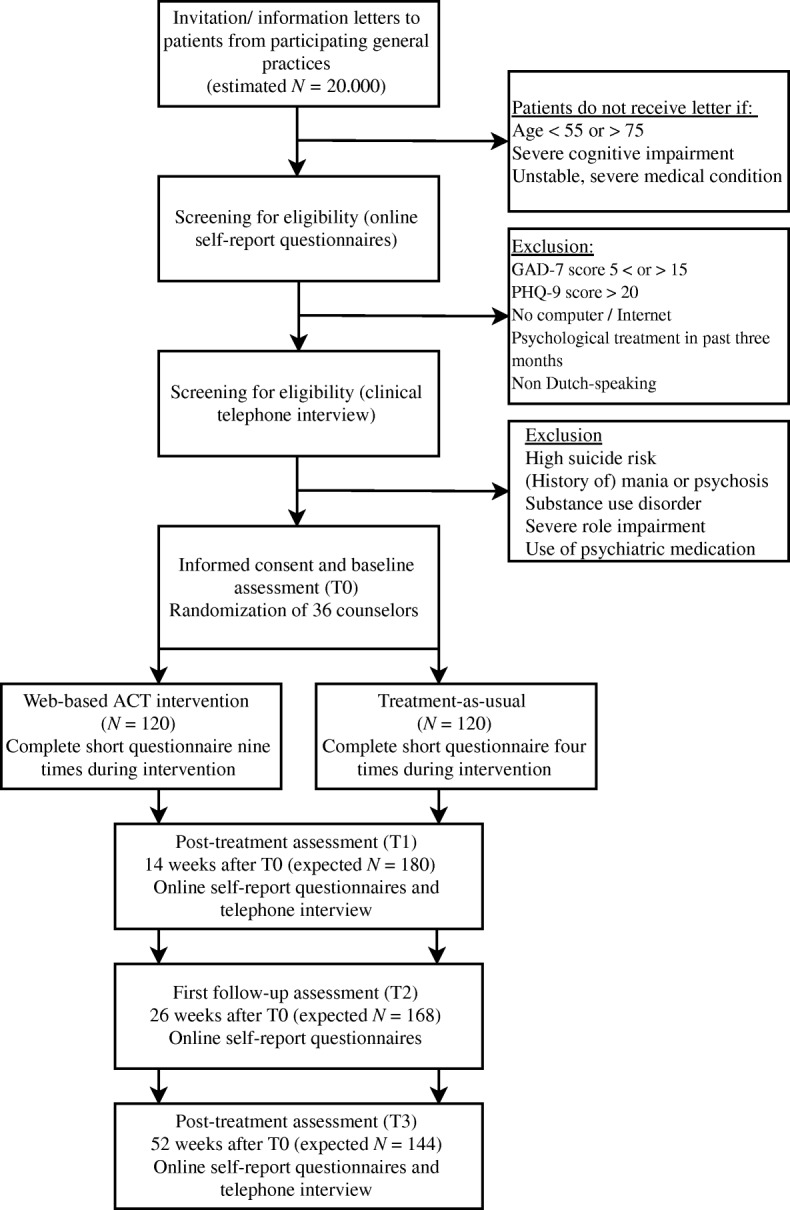
Diagram of patient flow through phases of the study

Figure 1 shows the flow chart of the study design. The SPIRIT Checklist and Figure are presented as Additional file 1 and 2 respectively. The study is approved by the medical ethical committee of the Leiden University Medical Center (LUMC; no. P16.248).

### Participants

A total of 240 participants will be included. In order for a person to be eligible to participate in the study, the following inclusion criteria have to be met: aged 55–75 years; presence of mild to moderate anxiety symptoms; access to Internet; mastery of the Dutch language; and the possibility and motivation to spend up to 30 min per day on the intervention. Potential individuals that meet any of the following exclusion criteria will be excluded from participation: Alzheimer’s disease or other severe cognitive impairments; unstable severe medical condition(s); severe anxiety or few anxiety symptoms; severe depressive symptomatology; having received psychological or psychopharmacological treatment (with the exception of stable use of benzodiazepines or SSRIs) within the last three months; severe role impairment in at least two areas of role functioning; high suicide risk; substance use disorder or a lifetime diagnosis of bipolar disorder or schizophrenia. The main inclusion criterion—severity of anxiety symptoms—is measured with the GAD-7. This questionnaire uses well-established cut-off points for mild, moderate, and severe anxiety symptoms [48]. These cut-offs point are used to identify our study population: people that score between 5 (cut-off point for mild anxiety) and 15 (cut-off point for severe anxiety) are considered to experience mild to moderately severe anxiety complaints. The information in the participants’ medical record at the general practice is used to determine the presence of a severe unstable medical condition, severe cognitive impairment, and a lifetime diagnosis of bipolar disorder or schizophrenia.. The PHQ-9 [49] is used to assess depressive symptomology (a score of ≥ 20 indicates severe symptoms and excludes people from participation). Role impairment is assessed with the Sheeran Disability Scale (SDS) [50]. A score of ≥ 8 on two of the three subscales is indicative of severe role impairment. Participants are screened for high suicide risk, substance use disorders, and a lifetime diagnosis of bipolar disorder or schizophrenia with the M.I.N.I.-Plus [51].

### Sample size

Cluster randomization will be applied at the level of the participating mental health counselors. Assuming a mean cluster size of five patients per counselor, an intraclass correlation coefficient of 0.01 [52] and a coefficient of variation of 0.30 [53], 18 mental health counselors in each intervention arm and 90 participants per study arm are needed to detect a between group difference on the GAD-7 with a medium effect size (d = 0.45) with an alpha of 0.05 (two-tailed) and a power of 0.80. Compensating for an anticipated dropout of 25% (based on trials similar to the proposed study with regard to either the treatment approach [40, 54] or the study population [26]), 240 participants will have to be included at baseline. The endpoint for this sample size is 14 weeks. In the most recent and comprehensive Cochrane meta-analysis of 101 studies of media-delivered CBT for anxiety disorders in adults, moderate-quality evidence showed medium effect sizes for CBT compared to control conditions (d = 0.67; 95% confidence interval = 0.55–0.80) [23]. These effects were somewhat smaller than those reported in other recent reviews [17, 19–22] and also smaller than the large effect sizes for Internet-based ACT (0.80 and 0.87) in the study of Fledderus et al. [40] and for Internet-based CBT for anxiety complaints in older adults (1.43) in the study of Dear et al. [26]. These last two studies both compared active treatment to a waiting list control condition. Furthermore, Pots et al. reported an effect size of 0.41 for web-based ACT (with e-mail support) compared to an active control group (expressive writing) [41]. Based on these studies, a moderate effect size of d = 0.45 is estimated for the difference between the ACT online intervention and treatment-as-usual.

### Procedure

#### Recruitment of general practices

General practices will be recruited throughout the Netherlands, with a focus on the Leiden and the Hague area. Both general practitioners and the mental health counselors working at the general practices will receive a printed invitation to participate in the study, after which they will be contacted by email and phone to recommend participation. When a practice (both GP and mental health counselor) agrees to participate, the mental health counselor will be randomized. A total of 36 mental health counselors will be included. Randomization is performed by an independent researcher, who is informed by the main researcher when a group of four mental health counselors have been enrolled. The mental health counselors are informed about their group allocation by the main researcher and are invited to attend a training in the treatment method they will apply during the study.

#### Recruitment of participants

A data manager will visit the participating general practices to assist the general practitioner in the selection of patients aged 55–75 years that are eligible to receive an invitation letter for the study. Patients whose medical records mention a lifetime diagnosis of bipolar disorder or schizophrenia, a severe unstable medical condition, Alzheimer’s disease, or other severe cognitive impairments will not receive an invitation. All other patients aged 55–75 years do receive an invitation letter. The invitation letter from the GP to the patients contains information about anxiety complaints and explains the aim, design, and procedure of the study. Furthermore, the letter refers to the study website. On this website, potential participants can read more extensive information about the study and indicate their interest to participate. After doing so, they will be asked to fill in the GAD-7 and PHQ-9 and to indicate whether they have received psychological treatment for emotional problems during the last three months, have Internet access, have sufficient time for treatment, are able to communicate in Dutch, and are aged 55–75 years. If based on these answers patients are still eligible to participate, they are asked if they are willing to be interviewed by phone to determine whether they fulfill all inclusion criteria. They are asked to fill in their telephone number and to indicate on which day they prefer to be called. Within ten days they will be phoned by a research assistant, who will administer the SDS and M.I.N.I.-Plus. Furthermore, they will be asked about their medication use. Patients will also be given the opportunity to ask any remaining questions about the aim of the study and the study procedures. The interviewer will discuss his/her diagnostic findings from the M.I.N.I-Plus with a senior clinical psychologist; subsequently the interviewee is informed by mail about inclusion or exclusion. When patients are eligible, they will receive a link to an online informed consent form and the baseline questionnaires (T0). Ineligible patients will be referred to their general practitioner in case they want help for their complaints. Once eligible patients have filled in the informed consent form and the baseline questionnaires, the mental health counselor at their general practice will be informed about study participation and they will be invited for a first appointment. In addition to the mass mailing, general practitioners and mental health counselors will recommend participation to patients aged 55–75 years that attend the practice with anxiety complaints during the inclusion period. They will inform these patients about the study and refer them to the study website if they want to participate. These patients then follow the screening and inclusion procedure as described above.

#### Procedure during study participation

During the intervention, participants will fill in a short questionnaire assessing potential mediator variables several times. At these moments they will also complete the GAD-2 [55] and PHQ-2 [56] to measure respectively anxiety and depression symptoms (the combination of PHQ-2 and GAD-2 is also known as the PHQ-4 [57]). They will also be asked whether they have had recurrent thoughts about death or hurting themselves in the past week, using an item of the PHQ-9 [49]. When participants have had suicidal thoughts in the past week, they will be recommended to discuss this with their mental health counselor at the general practice. Furthermore, their mental health counselor will be notified about the presence of suicidal thoughts by the researchers and are asked to contact the participant. Participants in the experimental condition complete this questionnaire at the end of every online lesson (nine times in total). Participants in the control condition, receive an email with a link to this questionnaire after every session with the mental health counselor (a total of four times).

Both interventions are delivered in a timespan of maximum 12 weeks, after which participants receive an e-mail with a link to the online self-report questionnaires of the post-treatment assessment (T1). T1 also includes a clinical interview by telephone conducted by a research assistant. The follow-up assessments take place six (T2) and twelve months (T3) after baseline. Both these assessments consist of self-report questionnaires and T3 also includes a clinical telephone interview. Figure 1 shows an overview of the patient flow through the phases of the study.

#### Prevention of attrition

Although available research shows that the effect of eHealth interventions does not depend on age and that older adults are even more adherent than younger adults [58], several precautions will be taken to reduce dropout and heighten compliance throughout this study. If participants fail to complete study assessments or intervention assessments, motivational reminders will be send repeatedly by email. Furthermore, individuals who complete all study assessments will take part in a lottery for gift vouchers worth €50, in order to stimulate them to complete all study assessments (lotteries will take place every year during the data collection period). If participants drop out or stop using the intervention, they will be asked for the reason(s) why they decided to quit the intervention and/or study.

### Treatments

#### Experimental condition

The online intervention “Living to the Full” is an adaptation of the similarly titled ACT-based self-help book [59] (English version: [60]). The effectiveness of this web-based intervention (both with and without support from a counselor) in reducing depression and associated anxiety in adults has been established in RCTs [21, 40, 41, 61]. A pilot study in which ten older adults worked through the online module, resulted in some age-matched adaptations of the layout and text of the intervention. The online module consists of nine lessons that are ideally completed in a maximum of 12 weeks. The lessons are based on the six core processes of ACT that aim to promote psychological flexibility: acceptance; cognitive defusion; contact with the present moment; self as context; values; and committed action. Each lesson contains information in both text and video form about the relationship between the ACT processes and emotional wellbeing, experiential exercises, metaphors, and motivational exercises. Furthermore, participants are instructed to practice mindfulness for 10–15 min on a daily basis. The intervention provides them with audio files that guide them through the mindfulness exercises. Each week, participants receive two or three standardized motivational text messages, to increase motivation and adherence. In addition to the online self-help module, participants will have four face-to-face sessions with the mental health counselor at their general practice to increase motivation, evaluate their progress, and discuss problems. For these sessions, a protocol has been developed. To measure adherence to the protocol, mental health counselors will complete a short online questionnaire after every face-to-face session which asks them to give a brief summary of the session. The mental health counselors in this condition will receive training from an experienced clinical psychologist to get acquainted with ACT, the protocol, and the online environment in which their patients will be working. During the study, the mental health counselors will have two supervision sessions from a clinical psychologist.

#### Control condition

Treatment as usual will be provided as optimized care as usual. Participants will be offered four face-to-face sessions with the mental health counselor at the general practice. The treatment follows the guideline for anxiety complaints as developed by the Dutch College of General Practitioners. This guideline prescribes psycho-education and, if necessary, short-term CBT or problem-solving therapy. A manual for the treatment older adults with anxiety complaints has been developed by the researchers. The manual comprises 12 small interventions. Each intervention focuses on either a common form of anxiety (worrying, panic symptoms, social anxiety), cognitive or behavioral aspects typical of most anxiety complaints (catastrophic thinking, avoidance), or consequences of anxiety (sleeping problems, physical tension). All interventions consist of psycho-educative texts and exercises based on CBT. During the first session, the mental health counselor and participant make an overview of the psychological complaints of the participant. Based on this inventory, one or more of the small interventions are selected to form the basis of the next three sessions. In order to prevent treatment diffusion, the delivery of interventions resembling the web-based interventions (i.e. eHealth interventions in general and ACT and mindfulness-based interventions in particular) are not allowed. Mental health counselors will complete a short online questionnaire that measures treatment protocol adherence after every session. Mental health counselors in this condition will follow a training from an experienced clinical psychologist, to practice with the protocol. They will also be supervised during the study.

### Assessments

Table 1 depicts an overview of the assessment instruments that will be used throughout the study. All questionnaires—except for the M.I.N.I.-Plus and SDS—will be conducted online. The M.I.N.I.-Plus and SDS are conducted by telephone.

**Table 1:** Overview of assessments during the study

Assessment	Screening	T0 Baseline	During intervention	T1 Post-intervention (14 weeks after T0)	T2 Follow-up (26 weeks after T0)	T3 Follow-up (52 weeks after T0)
GAD-7	X	X	–	X	X	X
PHQ-9	X	X	–	X	X	X
PHQ-4 (GAD-2 + PHQ-2)	–	–	X	–	–	
M.I.N.I.-Plus	X	–	–	X	–	X
SDS	X	–	–	X	–	X
CERQ	–	X	–	X	X	X
AAQ-II	–	X	–	X	X	X
MHC-SF	–	X	–	X	X	X
FFMQ-SF	–	X	–	X	X	X
EQ-5D-5 L	–	X	–	X	X	X
TIC-P	–	X	–	–	X	X
CSQ-8	–	–	–	X	–	–
Self-esteem,Mastery andSupport	–	X	–	–	–	–
Life events	–	X	–	–	–	–
Somatic problems	–	X	–	–	–	–
Demographics and other information	–	X	–	–	–	–
Treatment credibility and expectancy	–	–	X	–	–	–
Emotion regulation	–	X	X	X	X	X
Behavioral avoidance	–	X	X	X	X	X
Treatment expectancy	–	–	X	–	–	–
Self-efficacy	–	–	X	–	–	–
SRS	–	–	X	–	–	–

*GAD-7* General Anxiety Disorder 7, *PHQ-9* Patient Health Questionnaire 9, *PHQ-4* Patient Health Questionnaire 4,*GAD-2* General Anxiety Disorder 2, *PHQ-2* Patient Health Questionnaire 2, *M.I.N.I.-PLUS*, Mini-International Neuropsychiatric Interview-PLUS, *SDS* Sheehan Disability Scale, *CERQ* Cognitive Emotion Regulation Questionnaire, *AAQ-II* Acceptance and Action Questionnaire II, *MHC-SF* Mental Health Continuum Short Form, *FFMQ-SF* Five Facet Mindfulness Questionnaire Short Form, *EQ-5D-5 L* EuroQol 5 dimensions 5 levels questionnaire, *TiC-P* Trimbos/iMTA questionnaire for Costs associated with Psychiatric Illness, *CSQ-8* Client Satisfaction Questionnaire 8, *SRS* Session Rating Scale

### Primary outcome

#### Anxiety symptom severity

The primary outcome—severity of anxiety symptoms—will be assessed with the GAD-7 [48]. This questionnaire will be used to screen participants on anxiety symptoms and to measure change in these symptoms from baseline to post- and follow-up tests. The primary endpoint for this measure is the measurement 14 weeks after baseline (T1). The GAD-7 consists of seven items that are rated on a 4-point scale ranging from 0 (not at all) to 3 (nearly every day), e.g. “Over the last 2 weeks, how often have you been bothered by: feeling nervous, anxious or on edge.” Higher scores indicate more anxiety symptoms. Total scores are in the range of 0–21 and scores of 5, 10, and 15 are taken as the cut-off points for mild, moderate, and severe anxiety. Psychometric properties of the GAD-7 are adequate and the scale may also be used as a screener for panic disorder, social anxiety, and post-traumatic stress disorder [48, 55, 62]. The reliability and validity of a web-based Dutch translation of the GAD-7 have also been established [63].

Furthermore, during treatment, participants will fill in the GAD-2 [55] several times to measure anxiety symptom severity. The GAD-2 consists of the first two items of the GAD-7, which reflect core anxiety symptoms (“Feeling nervous, anxious, or on edge” and “Not being able to stop or control worrying”). Scores on the GAD-2 will be used in the mediation analyses. The GAD-2 is a reliable, valid, and sufficiently sensitive and specific instrument [55, 63, 64].

### Secondary outcomes

#### Depression symptom severity

Depression symptoms will be measured with the PHQ-9 [49]. This questionnaire will be used as a screener for depressive symptoms and to measure change in depressive symptomology from baseline to post- and follow-up tests. The questionnaire consists of nine items and includes the DSM-IV criteria for a major depressive disorder. The items are rated on a 4-point scale ranging from 0 (not at all) to 3 (nearly every day). Scores are in the range of 0–27 and cut-off points of 5, 10, 15, and 20 represent mild, moderate, moderately severe, and severe levels of depressive symptoms. The PHQ-9 is sensitive to change, has good sensitivity and specificity for detecting depressive disorders, and its psychometric properties are adequate [62, 64, 65]. In addition, depression symptoms will be measured with the PHQ-2 [56] at several times during treatment. The two items of the PHQ-2 (which correspond to the first two items of the PHQ-9) measure core depressive symptomology. The scores on these items will be used in the mediation analyses. The reliability, validity, and sensitivity to change of the PHQ-2 have been established [56, 57, 64].

#### Presence of psychiatric disorder

Presence of current and/or lifetime diagnosis of anxiety disorder (generalized anxiety disorder, panic disorder, agoraphobia, specific phobia, social phobia), obsessive-compulsive disorder, depressive disorder, post-traumatic stress disorder, and illness anxiety disorder will be determined by the M.I.N.I.-Plus [51]. Current substance abuse disorder and suicide risk as well as a lifetime diagnosis of psychotic disorder and bipolar disorder will also be assessed. The M.I.N.I.-Plus is the most widely used psychiatric structured diagnostic interview instrument in the world and has been validated against the Structured Clinical Interview for DSM diagnoses (SCID-P) and the Composite International Diagnostic Interview for ICD-10 (CIDI) [51]. Trained research assistants who are blind to the randomization scheme will conduct the M.I.N.I-Plus by phone. The M.I.N.I.-Plus will be administered during the screening (T0). At T1 and T3, only the modules for current anxiety disorders, depressive disorder, post-traumatic stress disorder, obsessive-compulsive disorder, and illness anxiety will be conducted. Minor adjustments will be made to the M.I.N.I.-Plus, so it corresponds with the criteria of the DSM-V. When uncertain of a diagnosis, the interviewers will have the opportunity to consult a psychiatrist or senior clinical psychologist, who is also blind to randomization status of the participants.

#### Functional impairment

The SDS [50] was developed to assess functional impairment in three inter-related domains: work/school; social life; and family life. The SDS measures the extent to which work/school, social life, and home life or family responsibilities are impaired by psychiatric symptoms on a 10-point visual analogue scale. The three items can be summed into a single dimensional measure of global functional impairment that ranges from 0 (unimpaired) to 30 (highly impaired). A psychometric analysis in a large sample of primary care patients demonstrated that the SDS has good psychometric qualities [50]. Research assistants that are blind to group allocation, will administer the SDS by telephone during the screening (T0) and at T1 and T3.

#### Cognitive emotion regulation

The subscales Rumination, Catastrophizing, Positive reappraisal, and Blaming yourself of the CERQ [66, 67] will be used to measure the extent to which participants use these cognitive coping strategies when confronted with adversities. The choice for these subscales is based on several studies that have demonstrated the association between these strategies and anxiety and depression symptoms [67–69]. The subscales consist of four items each. Higher scores on a subscale indicate that this cognitive coping strategy is more often used to regulate emotions. Psychometric properties of the Dutch CERQ are adequate [68, 70].

#### Experiential avoidance

The Acceptance and Action Questionnaire (AAQ-II) [71, 72] is a uni-dimensional measure [72, 73] that assesses experiential avoidance: the unwillingness to remain in contact with aversive private experience and behaviors aimed at altering these experiences or the events that elicit them. The AAQ-II originally contained ten items, but a study showed that a seven-item version has better psychometric qualities [72]. Items are scored on a 7-point scale ranging from 1 (never true) to 7 (always true). Higher scores reflect higher levels of experiential avoidance. The reliability and validity of a Dutch translation of the AAQ-II has been established in a sample of moderately depressed and anxious individuals [73].

#### Positive mental health

The Mental Health Continuum-Short Form (MHC-SF) [74] is a 14-item self-report questionnaire for positive mental health assessment. The questionnaire consists of three subscales, corresponding with the three dimensions of positive mental health: three items for emotional wellbeing; six items for psychological wellbeing; and five items for social wellbeing. Items are rated on a 6-point scale ranging from 0 (never) to 5 (every day). Confirmatory factor analysis (CFA) confirmed the three-factor structure of a Dutch translation of the MHC-SF. Results from the same psychometric study also revealed high internal and moderate test–retest reliability for the Dutch MHC-SF [74].

#### Mindfulness

The Five Facet Mindfulness Questionnaire- Short Form (FFMQ-SF) [75] will be used to measure mindfulness, defined as the ability to bring one’s attention to experiences in the present moment in a non-judgmental way [76]. The questionnaire contains 24 items that measure five facets of mindfulness: observing; describing; acting with awareness; non-judging; and non-reactivity. Items are scored on a 5-point scale ranging from 1 (never or very rarely true) to 5 (very often or always true). A psychometric study showed that the FFMQ-SF is a sensitive, reliable, and valid instrument. Furthermore, the five-factor structure of the questionnaire was confirmed in this study [75].

#### Generic health status and quality of life

The EuroQol-5 Dimensions-5 Levels questionnaire (EQ-5D-5 L) measures general quality of life using five dimensions (i.e. mobility, self-care, usual activities, pain/discomfort, and anxiety/depression) [77]. Each dimension has five response categories, describing the severity of problems. A total of 3125 unique health states can be defined, by combining the responses for the five dimensions into a five-digit number (ranging from “11111” meaning no problems at all to “55555” meaning extreme problems in all five dimensions) [78]. The EQ-5D-5 L is an adaption of the EQ-5D [79]. The EQ-5D consists of the same five dimensions but has only three response categories per dimension. Evidence suggests that the EQ-5D has limited responsiveness to changes in health, partly caused by ceiling and floor effects. Studies show that compared to the EQ-5D, the EQ-5D-5 L has smaller ceiling and floor effects, and improved reliability and discriminating ability [80, 81].

#### Costs associated with psychiatric illness

For calculating the total direct medical costs, the Trimbos/iMTA questionnaire for Costs associated with Psychiatric Illness (TiC-P) [82] will be used. The TiC-P measures utilization of medical treatment, such as the number of contacts with the general practitioner and multiple other care providers (e.g. medical specialists and paramedics) during the last four weeks, as well as medication use and loss of productivity at (voluntary) work. The costs will be calculated using the Dutch guidelines for cost calculations in healthcare. Reference unit prices of the corresponding healthcare services will be applied. A psychometric study demonstrated that the Dutch version of the TIC-P is a feasible, reliable, and valid tool for assessing care consumption and productivity loss in patients with mild to moderate psychiatric problems [83].

#### Client satisfaction

The Client Satisfaction Questionnaire-8 (CSQ-8) [81] is an eight-item instrument that is designed to measure client satisfaction with services. The items for the CSQ-8 were selected based on mental health professionals’ ratings of a number of items that could be related to client satisfaction and a subsequent factor analysis. The CSQ-8 is uni-dimensional, yielding a homogeneous estimate of general satisfaction with services. The questions are answered on a 4-point scale in the range of 1–4, but each question has different labels attached to these values. Higher scores indicate higher client satisfaction. The psychometric qualities of the Dutch translation of the CSQ are adequate [84, 85].

### Moderator variables

#### Self-esteem, mastery, and support

Starting from existing instruments, Bovier, Chamot, and Perneger used factor analyses to develop four brief scales for the assessment of self-esteem, affective social and confident/problem-solving social support [86]. All 12 items are answered on a 5-point Likert scale, with higher scores representing higher levels of the four measured constructs. All four scales have been found to demonstrate good internal and construct validity [86].

#### Life events

A self-developed questionnaire will be used to measure negative life events and associated distress. Participants are asked if they have experienced negative life events in the past six months and/or earlier in their life. If they indicate that they have experienced such (an) event(s), they are asked to rate them on an 11-point scale (ranging from 0 [not at all] to 10 [extremely]) to what extent these experiences currently still evoke strong negative feelings.

#### Co-morbid somatic problems

Co-morbid physical problems will be measured with a self-developed questionnaire listing 25 (chronic) conditions. This list is based on information from Statistics Netherlands. Participants will also be asked to rate to what extent their somatic problem(s) interferes with their current daily functioning on an 11-point scale ranging from 0 (not at all) to 10 (extremely).

#### Socio-demographics and other information

Using a self-developed questionnaire, the following socio-demographic information and additional information will be collected: age; gender; nationality; marital status; living conditions; education; work status; computer usage; and Internet usage.

#### Treatment credibility and expectancies

Participants’ expectations of the treatment will be measured with a questionnaire derived from the Treatment Credibility Questionnaire (TCQ) from Borkovec and Nau [87]. In this study, the version as developed by De Jong et al. (Risk models for negative treatment outcomes in psychiatric outpatients: predicting end state functioning and rate of change using classification and regression trees (CART) and multilevel modeling, unpublished) will be used. This version combines an adaptation of the TCQ [88] with one item (“How much improvement in your symptoms do you think will occur”) from the Credibility Expectancy Questionnaire [89]. The questionnaire consists of seven items that are scored on a 7-point rating scale, ranging from 1 (not at all) to 7 (extremely). The questionnaire consists of two factors: Expectancies and Credibility. The version that will be used in this study has an internal consistency of 0.89 for the Expectancies subscale and 0.84 for the Credibility subscale [90]. This questionnaire will be completed after participants have had the first session with the mental health counselor at the general practice.

### Mediator variables

#### Emotion regulation

A self-developed questionnaire measures the use of the following emotion regulation strategies: distraction; reappraisal; acceptance; rumination/worry; and suppression. Adaptive strategies (distraction, reappraisal, acceptance) are consistently more strongly related with reduced negative affect and maladaptive strategies (rumination/worry and suppression) with enhanced negative affect in laboratory studies [91] and psychopathology in clinical studies [92]. In addition, participants will be asked whether they used problem-solving and attempted to change the stressful situation or contain its consequences. Although problem-solving responses are not directly aimed at regulating emotions, they can have beneficial effects on emotions by modifying or eliminating stressors [92]. Participants will report on the extent to which they have engaged in each of these emotion-regulation strategies on a 6-point scale ranging from 0 (never) to 6 (always). The questionnaire consists of a total of seven items.

#### Behavioral avoidance

Since both interventions stimulate participants to limit their avoidance behavior, this factor will be included in the mediation analyses. Behavioral avoidance is measured with one item (i.e. “In the past week my anxiety caused me to avoid situations and/or activities”), that participants rate on a 6-point-scale ranging from 0 (never) to 6 (always).

#### Treatment expectancy

Treatment expectancy is measured with one item: “How confident are you that the course will be helpful in reducing your anxiety complaints?” Participants rate this question on a 7-point scale ranging from 1 (not at all) to 7 (very confident). Several studies have shown the impact of client expectancies on treatment effect. Evidence suggests that eliciting hope and positive expectations about the effect of the treatment is a crucial factor in many psychotherapies [93–96].

#### Self-efficacy

Preliminary results suggest that the outcome of psychological interventions may be mediated by patient’s self-efficacy with regard to the treatment (i.e. one’s judgment of the capability to successfully participate in and complete the treatment) [97, 98]. Self-efficacy with regard to therapy is measured with one item: “How confident are you that you will do what is required to successfully follow and complete this course?” Participants rate this question on a 7-point scale ranging from 1 (not at all) to 7 (very confident*).*

#### Session Rating Scale

The Session Rating Scale (SRS) [99] will be used to measure the working alliance between the participants and their mental health counselor. The items assess four aspects of the working alliance: the relational bond (the degree to which one feels heard, understood, and respected); the degree to which desired goals and topics of the individual were discussed; an evaluation of the therapist’s approach or method that was used; and an evaluation of how the individual perceives the session overall. Instead of using a visual analogue scale, an 11-point scale will be used to answer each of the four items, with “0” depicting the most negative response and “10” depicting the most positive response. The SRS was shown to have high test–retest and internal consistency reliability, as well as acceptable validity [100, 101]. The SRS will be completed after every face-to-face session.

### Statistical analysis

All analyses will use intention-to-treat principles and a two-tailed alpha of 0.05 for significance testing. Cluster randomization at the level of mental health counselors (instead of individual patients) results in a lack of independence for the outcomes of patients receiving treatment from the same counselor. If clustering and dependence of outcome are ignored, this could lead to underestimation of standard errors and regression coefficients [102]. Therefore, multilevel regression analysis will be used to examine the treatment effect on the primary and secondary outcome measures. Multilevel analysis allows modeling of the variability of the outcome measures within clusters and analysis of a repeated-measure design with missing data. In this cluster randomized RCT, three levels can be distinguished: (1) repeated measures within patients; (2) treatment allocation; and (3) mental health counselors. To evaluate the intervention effect, a twofold analysis will be conducted. First, using a mixed-model analysis with treatment as a dummy variable and the dependent variable on baseline as covariate it will be examined whether conditions differ across time. In addition, a subsequent mixed-model analysis without the dependent variable at baseline as covariate will analyze at which particular time point conditions differ as indicated by the interaction effect of treatment × time. Missing data will be imputed using a model-based approach. Covariance will be estimated using an unstructured covariance matrix.

To examine moderators of the treatment outcome, longitudinal multi-level regression analysis will be performed. Potential moderators will be entered individually in the model. Interaction effects will be investigated (e.g. time × condition × potential moderator). Bivariate latent difference score models will be used for the mediation analysis. This type of analysis is recommended for repeated measurements and multiple mediators, because they allow for a more dynamic approach to mediation, by assessing changes in multiple variables and their interrelations over time [103–105]. Cost-effectiveness analyses will be conducted in agreement with the CHEERS statement [106] and will be determined by relating the difference in healthcare costs (measured with the TiC-P) to the between-group difference in reliable change on GAD-7 scores (cost-effectiveness analysis) and quality-adjusted life-years (QALYs) gained based on the EQ-5D-5 L using the Dutch tariffs (cost–utility analysis). Productivity losses in paid work will be included in the economic evaluation and measured by assessing work-loss days due to absenteeism and work cutback days (presenteeism) while at work but not feeling well. All cost prices will be indexed for the appropriate reference year (likely to be 2017) but not discounted because the study’s time horizon does not exceed the period of a single year. Stochastic uncertainty in the incremental cost-effectiveness ratios (ICERs) will be handled using non-parametric bootstraps (2500 replications), plotted on the ICER plane and depicted in an ICER acceptability curve. Sensitivity analyses will focus on the friction cost versus human capital method and on main cost-drivers.

## Discussion

The proposed study will evaluate the (cost-)effectiveness of the Internet-based ACT intervention ‘Living to the Full’ for older adults with anxiety complaints in a RCT. This study has several strengths. First, the trial is unique in this field of research. It differs from the majority of studies in older anxious adults with regard to therapy approach (ACT instead of CBT), the delivery of the investigated intervention (Internet-based instead of face-to-face) and the study population (people with anxiety complaints instead of anxiety disorders). The moderation and mediation analyses form a second strength. These analyses will provide insight into respectively the effect of personal characteristics on treatment outcome and the processes through which the intervention achieves its effects. Including such analyses broadens the scope of the study. The trial is not merely focused on answering the question if “Living to the Full” is more or less effective than treatment-as-usual. Because of the optimized active control condition, it is plausible that both treatments will result in a significant reduction in symptomology and related measures. In this scenario, the moderation and mediation analyses will still be of value in exploring if certain subgroups of patients benefit from one of the interventions in particular and if the processes through which the treatments achieved their effects differ. This sort of knowledge can contribute to a more personalized treatment approach by taking into account moderating factors and an increased effectiveness of interventions by refining and intensifying those components that focus directly on the mediators [107]. Another strength of this study is related to the fact that treatment-as-usual is delivered as optimized care as usual. Because participants in the control condition will receive appropriate treatment, a 12-month follow-up is defendable, allowing to investigate the long-term (cost-)effectiveness of the “Living to the Full” intervention compared to treatment as usual. A final strength of this study is its recruitment method. All patients aged 55–75 years from participating general practices are actively invited to participate in the study and are able to register online. Compared to studies in which only patients that visit their general practitioner with certain complaints are informed about a study, this method reaches a wider population and lowers the threshold for registering for participation.

The study also has some limitations. First, the online nature of the screening procedure and intervention might limit the study sample. It excludes people that are not proficient with computers. These people might share certain characteristics (e.g. older age, lower education levels), which reduces the representativeness of the sample. Second, the study might encounter difficulties in recruiting enough participants. Recruiting older adults for study participation is challenging [108]. Furthermore, due to the cluster-randomized design, more participants are needed to obtain equivalent statistical power as compared to an individually randomized trial [47]. However, this study uses a mass mailing as the main recruitment method, which has proven to be one of the most successful strategies to recruit older adults [108].

To conclude, the proposed study will evaluate an ACT online self-help program combined with face-to-face contacts with the mental health counselor at the general practice for older adults with anxiety symptoms. Since subclinical anxiety is highly prevalent in this age group, (cost-)effective, low-threshold interventions are needed. The proposed study complies with this request. When “Living to the Full” proves to be effective for this patient group, implementation of the intervention in general practices in the Netherlands will follow.

### Trial status

Patient recruitment started November 2017. Recruitment is expected to be completed December 2018.

## Additional files

Additional file 1: Spirit Checklist. (DOC 121 kb)

Additional file 2: Spirit Figure. (PDF 118 kb)

## Abbreviations

AAQ-II: Acceptance and Action Questionnaire II
ACT: Acceptance and Commitment Therapy
CBT: Cognitive behavioral therapy
CERQ: Cognitive Emotion Regulation Questionnaire
CSQ-8: Client Satisfaction Questionnaire 8
EQ-5D-5 L: EuroQol 5 dimensions 5 levels questionnaire
FFMQ-SF: Five Facet Mindfulness Questionnaire Short Form
GAD-2: General Anxiety Disorder 2
GAD-7: General Anxiety Disorder 7
M.I.N.I.-PLUS: Mini-International Neuropsychiatric Interview-PLUS
MHC-SF: Mental Health Continuum Short Form
PHQ-2: Patient Health Questionnaire 2
PHQ-4: Patient Health Questionnaire 4
PHQ-9: Patient Health Questionnaire 9
SDS: Sheehan Disability Scale
SRS: Session Rating Scale
TiC-P: Trimbos/iMTA questionnaire for Costs associated with Psychiatric Illness
