# Supplementary figures and images for: An Internet-based Acceptance and Commitment Therapy intervention for older adults with anxiety complaints: study protocol for a cluster randomized controlled trial
Source: Trials. 2018 Sep 17;19:502. doi: 10.1186/s13063-018-2731-3 (PMC6142346; doi:10.1186/s13063-018-2731-3)

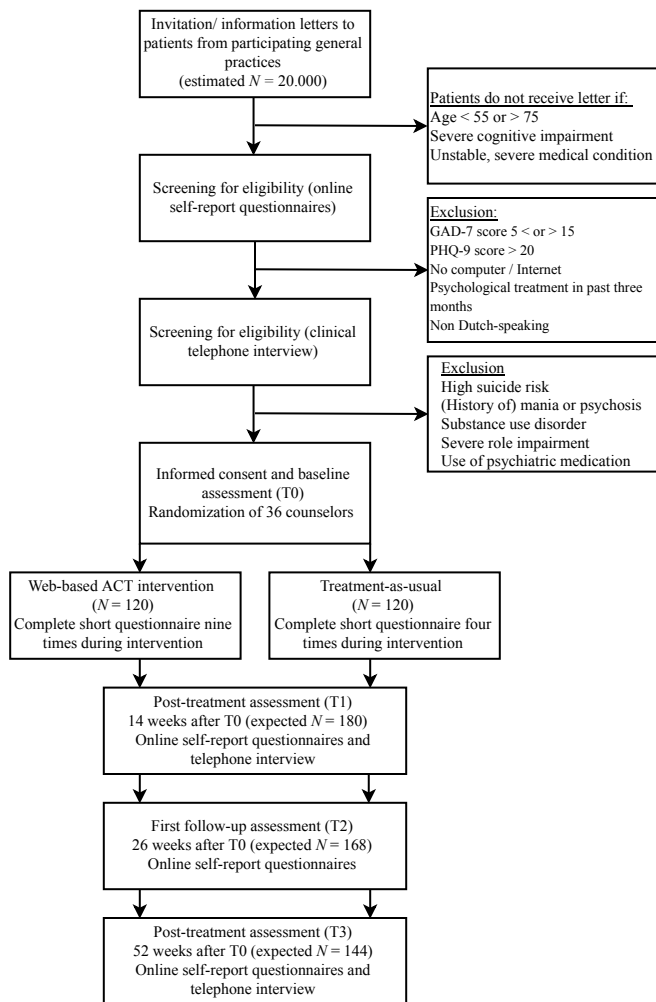

Figure 1. Diagram of patient flow through the phases of the study

Supplement: Supplementary file 2 — Spirit Figure. (PDF 118 kb) [file 13063_2018_2731_MOESM2_ESM.pdf]
